# Antibody-dependent cellular cytotoxicity-null effector developed using mammalian and plant GlycoDelete platform

**DOI:** 10.1038/s41598-022-23311-9

**Published:** 2022-11-08

**Authors:** Cho Eun Kang, Seungeun Lee, Taeyoung Ahn, Dong Hye Seo, Byoung Joon Ko, Minkyu Jung, Jinu Lee, Joo Young Kim, Woo Taek Kim

**Affiliations:** 1 Department of Pharmacology and Brain Korea 21 Project for Medical Science, Yonsei University College of Medicine, 50-1 Yonsei-Ro, Seodaemun-Gu, Seoul, Republic of Korea; 2 Department of Systems Biology, College of Life Science and Biotechnology, Yonsei University, 50 Yonsei-Ro, Seodaemun-Gu, Seoul, 03080 Republic of Korea; 3 School of Biopharmaceutical and Medical Sciences, Sungshin Women’s University, Seoul, 02844 Republic of Korea; 4 Department of Oncology, Yonsei University College of Medicine, Seoul, Republic of Korea; 5 College of Pharmacy, Yonsei Institute of Pharmaceutical Sciences, Yonsei University, 85 Songdogwahak-Ro, Yeonsu-Gu, Incheon, 21983 Republic of Korea

**Keywords:** Antibody therapy, Cancer immunotherapy, Plant biotechnology

## Abstract

Cancer therapy using immune checkpoint inhibitor antibodies has markedly shifted the paradigm of cancer treatment. However, methods completely eliminating the effector function of these signal-regulating antibodies is urgently required. The heterogeneity of glycan chains in antibodies limits their use as therapeutic agents due to their variability; thus, the development of uniform glycan chains is necessary. Here, we subjected the anti-programmed cell death protein (PD)-1 antibody nivolumab, a representative immune checkpoint inhibitor, to GlycoDelete (GD) engineering to remove the antibody-dependent cellular cytotoxicity (ADCC) of the antibody, leaving only one glycan in the Fc. Glyco-engineered CHO cells were prepared by overexpressing endo-β-N-acetyl-glucosaminidase (Endo T) in CHO cells, in which N-acetyl-glucosaminyl-transferase I was knocked out using Cas9. GD IgG1 nivolumab and GD IgG4 nivolumab were produced using GD CHO cells, and glycan removal was confirmed using mass spectrometry. Target binding and PD-1 inhibition was not altered; however, ADCC decreased. Furthermore, the IgG4 form, determined to be the most suitable form of GD nivolumab, was produced in a plant GD system. The plant GD nivolumab also reduced ADCC without affecting PD-1 inhibitory function. Thus, CHO and plant GD platforms can be used to improve signal-regulating antibodies by reducing their effector function.

## Introduction

Antibodies to immune checkpoints, such as programmed cell death protein 1 (PD-1), are becoming increasingly important in cancer treatment^1, 2^. PD-1 is an inhibitory immunomodulatory receptor that is highly expressed on tumor-specific T cells and inductively expressed on activated T, natural killer, B lymphocytes, macrophages, dendritic cells, and monocytes^3^. Blocking the PD-1 pathway restores the function of exhausted T cells, resulting in substantial antitumor activity^4^. Considering the trend of regulating the immune activity of various immune cells, inhibition of PD-1 activity may be useful for activating more diverse immune cells.

When using immunomodulatory anticancer drugs such as anti-PD-1 antibody, effector function such as complement-dependent cytotoxicity (CDC) and antibody-dependent cellular cytotoxicity (ADCC) of the antibody results in the death of T cells with anticancer immune activity; therefore, these toxicities must be eliminated^5, 6^. Most anti-PD-1 antibodies are of the IgG4 isotype and contain the S228P mutation to eliminate Fab exchange^7^, which has similar effector-binding properties as natural IgG4 but with reduced ADCC and “null” complement-dependent cytotoxicity^5, 8^. However, this isotype maintains high affinity for FcγRI^9^, which can cause T cell death at the high therapeutic dose^6^. Moreover, the high affinity of IgG4 for FcγRI may negatively affect the efficacy of PD-1 therapy^5^. Indeed, Dahan et al. reported that engagement of FcγRs reduced the anti-tumor activity of an anti-PD-1 antibody by eliminating CD8^+^ tumor-infiltrating lymphocytes via ADCC in a FcγRI-dependent manner^10^. Tumor-associated macrophages quickly removed these anti-PD-1 antibodies from T cells, thus inactivating them^11^. Therefore, a method is needed to remove the effector function of an immuno-oncology agent prepared from the Fc skeleton of an antibody.

The glycan chains of an antibody greatly contribute to the function and stability of the antibody^12, 13^. The ability of the Fc region to determine the complement or effector function of immune cells is determined by the binding ability between the FcγR receptor of immune cells and glycan structure of the Fc region of an antibody^9, 14^. These sugar chains can be modified through glyco-engineering of CHO cells, an antibody-producing cell line, as an important method for determining or improving the quality of pharmaceuticals^15^. Atezolimumab is a target antagonistic antibody and IgG1 antibody in which asparagine, an amino acid to which glycan binds, is substituted with alanine, and effector function is removed by removing all glycans^16^. However, atezolimumab severely aggregates because of glycan^17, 18^ removal and forms an antibody against it, suggesting that a method is needed for glycan control rather than for removing all glycans.

GlycoDelete (GD) is a glyco-engineering technology that reduces binding to FcγR by leaving only one N-acetyl-glucosamine (GlcNAc) on the antibody^19, 20^. In addition to being generated by the sequential action of several enzymes, glycan chains heterogeneously generated through the activity of different enzymes may interfere with uniformity of the drug^16, 21^. GD can maintain a drug's effect constant by homogenizing the glycan chain as well as by removing it^20^. However, it remains unclear whether Fab affinity is affected by GD or whether the same ADCC reduction effect of GD can be achieved when GD is applied to IgG4 and IgG1, respectively. When applying GD to the anti-PD-1 antibody, it is necessary to verify whether T cell death caused by the anti-PD-1 antibody has been eliminated.

GD can also facilitate the production of biopharmaceuticals using plants^22^. The system used to produce proteins in plants is a promising next-generation bio-drug production platform with high economic efficiency and safety for mass production of recombinant protein drugs^23, 24^. However, there is concern regarding the use of plant proteins as injectable drugs because antibodies to plant-specific β-1,2-xylose and core α-1,3-fucose sugar chains are detected at a high rate in not only allergy sufferers but also the general public^23, 25^. ZMapp, which ended the outbreak of Ebola, is an antibody treatment produced from genetically modified tobacco in which plant-specific sugar chains have been removed^26^. Additionally, the β-glucobulusidase taliglucerase alfa, a treatment for Gaucher disease sold by Plotalix, has a high-mannose sugar chain that is also found in mammalian proteins^27^. Although it remains controversial whether human acute allergy is caused by plant-specific sugar chains^28^, removing plant-specific sugar chains clearly increases the drug safety and treatment preference. Plant GD technology can be used to remove these plant-specific sugar chains.

This study investigated whether the effector function of immune checkpoint inhibitor antibodies could be reduced by GD technology that would result in only one GlcNAc molecule in the Fc portion of the antibody. GD engineering was performed in CHO cells and in tobacco plants to establish the GD CHO cell and the GD plant systems. The immune checkpoint inhibitory functions and T cell death rate of GD nivolumab produced were compared to those of the prototype.

## Results

### Successful production of GD CHO-IgG1 and IgG4 nivolumab antibody

**Figure 1:**
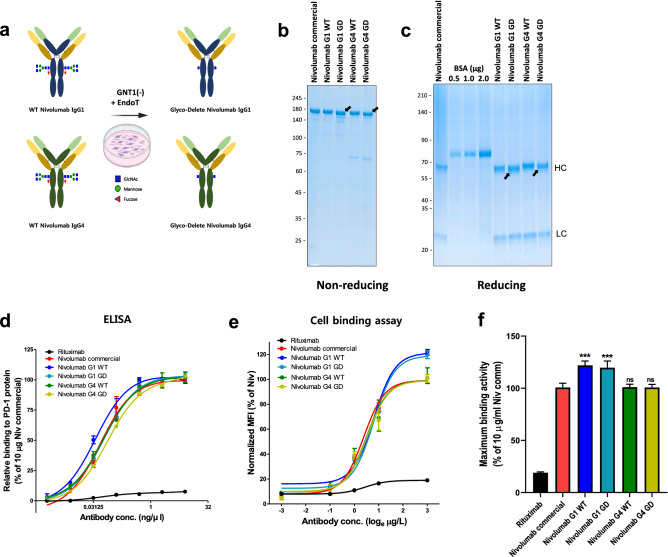
Production of CHO-GD nivolumab with IgG1 and IgG4 backbone (**a**) Schematic graphic of the GD process of nivolumab in the CHO cell system. (**b**) Nivolumab commercial, nivolumab IgG1 WT, nivolumab IgG1 GD, nivolumab IgG4 WT, and nivolumab IgG4 GD were subjected to SDS-PAGE under non-reducing and reducing conditions. BSA (0.5, 1.0, and 2.0 μg) was used as the standard. (**b**,**c**) CHO-nivolumab, CHO-nivolumab IgG1 WT, CHO-nivolumab IgG1 GD, CHO-nivolumab IgG4 WT, and CHO-nivolumab IgG4 GD were subjected to SDS-PAGE under non-reducing (**b**) and reducing (**c**) conditions. BSA (0.5 μg, 1.0 μg and 2.0 μg) was used as the standard. Binding affinity of each antibody (CHO-nivolumab, CHO-nivolumab IgG1 WT, CHO-nivolumab IgG1 GD, CHO-nivolumab IgG4 WT, CHO-nivolumab IgG4 GD, and rituximab) was analyzed via ELISA using recombinant human PD-1 protein **(d)** and via FACS using PD-1 expressed CHO cells **(e)**. Rituximab was used as a negative control for both analyses. **(d)** ELISA was performed with plates coated with recombinant human PD-1 protein, and the bound antibodies were detected with HRP-conjugated anti-human IgG antibody. **(e,f)** The binding capacities of the antibodies against PD-1-expressing CHO cell are sown in dose-dependent manner dependent (0.1 μg/mL, 0.3 μg/mL, 1 μg/mL, 3 μg/mL, and 10 μg/mL) **(e)** and the binding activity of each antibody at the maximum concentration (10 μg/ml) is summarized. **(f)** using flow cytometry and 2nd-anti human IgG-FITC. The mean fluorescence intensity (MFI) of FITC treated with nivolumab commercial (10 μg/mL) was normalized to 100%. The MFI of each antibody-treated cell was expressed as the relative %. ***p ≤ 0.001 compared to 10 μg/mL of nivolumab commercial. Rituximab was used as negative control in **(d)** and (**e,f)**.

The procedure for generating GD nivolumab from CHO cells is shown in Fig. 1a. In order to block the highly ordered glycosylation cascade initiated by GlcNAc transferase, we first removed GNTI enzyme via HITI Cas9 gene editing system to generate high-mannose form glycans. Then, Endo T, which cleaves high mannose glycans, leaving only one GlcNAc, was stably overexpressed through the lentivirus system. These two steps leave only one GlcNAc glycan in the Fc region of antibody. To examine the optimal GD-engineered Ig backbone that resulted in low effector function, we compared the IgG1 and IgG4 isotypes of nivolumab expressed in WT CHO and GD CHO cells. The SDS-PAGE was performed under non-reducing and reducing conditions (Fig. 1b,c) showed that the molecular weights of both the IgG1 GD and IgG4 GD nivolumab heterodimer complexes were slightly decreased (Fig. 1b). The decrease in molecular weight was only observed in the heavy chain, not in the light chain of IgG (Fig. 1c), indicating that the glycan in the heavy chain was removed by GD engineering. The removal of glycan from the heavy chain was evaluated via intact mass spectrometry of the GD IgG4 nivolumab Fc region after treatment with IdeS (a cysteine proteinase cleaving IgG to F(ab)_2_ and Fc/2 fragments), which proved that the glycan was modified based on the decreased molecular weight of Fc/2 fragment than expected molecular weight (Supplementary Fig. S2, Supplementary Table S1). Unexpectedly, the Fc/2 region showed two molecular weight peaks, one corresponding to the high-mannose form and one to the GlcNAc form. The molecular weight of GD IgG4 nivolumab (Fc/2) was reduced by 226.3 (high-mannose form) and by 1240.3 Da (one GlcNAc form). These data indicated that Endo T did not completely cleave the high-mannose glycan of the heavy chain.

Next, the binding affinity of the antibodies was measured by ELISA using recombinant human PD-1 protein (Fig. 1d). All five antibodies showed similar binding affinities at all doses of PD-1 protein regardless of the backbone and glycan. In order to evaluate the binding affinity of the antibodies against the PD-1 expressed on cells, PD-1-expressing CHO cells were used for FACS analysis (Fig. 1e,f). Interestingly, the nivolumab with IgG1 backbone showed higher binding activity than the nivolumab with IgG4 backbone, at the maximum antibody concentration (10 μg/mL). This result indicates that the IgG1 nivolumab possesses higher binding potency against the PD-1, compare to the IgG4 nivolumab (Fig. 1f).

These data demonstrate that GD nivolumab with the IgG1 and IgG4 backbone was produced and had similar or enhanced binding affinity compared to that of commercial nivolumab.

### Comparable PD-1-blockade activity and reduced CDC and ADCC efficacy of GD-nivolumab

**Figure 2:**
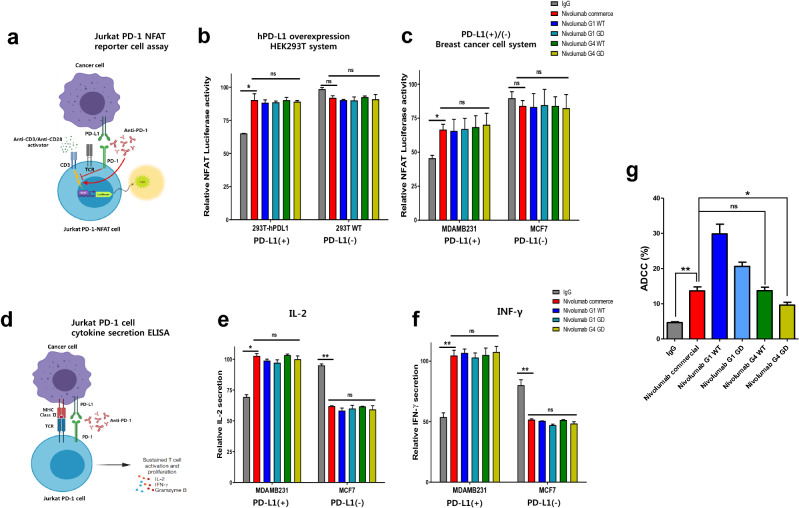
Comparable PD-1-blockade activity and reduced ADCC efficacy of CHO-GD nivolumab. Graphs of Jurkat PD-1 NFAT reporter cell system (**a**) and cytokine secretion of Jurkat PD-1 cells (**d**) were used to analyze the PD-1 blocking function of CHO-GD nivolumab. (**b,c**) PD-1 blocking activity of CHO-GD nivolumab (30 μg/mL) was confirmed via NFAT luciferase assay. Jurkat PD-1-NFAT cells were co-cultured with PD-L1 (+) (**b**) or PD-L1 (−) (**c**) cells and stimulated with an anti-CD3/anti-CD28 activator. Luciferase activity was measured 6 h after stimulation. (**e,f**) IL-2 (**e**) and IFN-γ (**f**) secretion activities of CHO-GD nivolumab. Jurkat PD-1 cells were co-cultured with breast cancer cell lines and treated with an anti-CD3/anti-CD28 activator and 30 μg/mL of each antibody. After 24 h of incubation, cell media were used for the ELISA. IgG was used as a negative control. (**g**) PD-1 expressing T cell cytotoxicity by CHO-GD nivolumab. ADCC performed by 10 μg/mL each antibody was analyzed using calcein-loaded Jurkat PD-1 cells and PBMCs from healthy donors as effector cells (1:3 target cell ratio: effector cell). Cell death rate was deducted from the live cells percentage, which was calculated by the numbers of calcein-containing cells 4 h after antibody treatment. WT IgG1 nivolumab and IgG were used for positive and negative control, respectively. Statistical significances were indicated as *p ≤ 0.05, **p ≤ 0.01, and ***p ≤ 0.001, respectively. ns, not significant.

To test PD-1 blockade activity, the Jurkat PD-1-NFAT luciferase reporter cell system (Fig. 2a) and cytokines secretion system such as IL-2 and IFN-γ from Jurkat PD-1 (Fig. 2d) were established^19, 20^. The luminescence of Jurkat-PD-1-NFAT-luciferase stable cells (Fig. 2b,c) and secretion of cytokines from Jurkat PD-1 cells (Fig. 2e,f) were used to monitor T cell activity after each antibody treatment. The full activity of Jurkat PD-1-NFAT luciferase reporter cells or cytokine release from Jurkat PD-1 cells was induced by an anti-CD3/CD28 activator, and the recovery of activity followed by treatment with 30 μg/mL of each antibody was measured based on the attenuated PD-L1 engagement. To validate the specificity of PD-L1-dependent PD-1 activation, WT HEK 293 T cell or human PD-L1-overexpressing HEK 293 T cells (Fig. 2b) and breast cancer cell lines MCF7 (PD-L1 (−)) or MDAMB231 (PD-L1 (+)) (Fig. 2c,e,f) were used. hPD-L1-overexpressing HEK293T cells (Fig. 2b) and MDAMB231 (Fig. 2b,e,f) showed lower luciferase activity and low cytokines secretion compared to those in WT HEK293T and MCF7 cells, respectively. Furthermore, significantly higher luciferase activity and cytokine secretion induced by the commercial nivolumab than that induced by IgG implies that the two systems are suitable for measuring the PD-1-blocking function of the antibodies. All five antibodies showed similarly increased luciferase activity and cytokine secretion, suggesting similar PD-1 blocking activities (Fig. 2b,c,e,f). Strangely, IgG treatment in PD-L1(−) cells led to higher luciferase activity than IgG treatment in hPD-L1-overexpressing HEK293T cells. However, no antibodies can increase the luciferase activity and IL-2 and IFN-γ secretion of PD-L1(−) cells than IgG. These data demonstrate that the PD-1 blocking activity of the 4 types of nivolumab was similar to that of commercial nivolumab, and thus the functional efficacy of nivolumab was not altered by GD engineering.

Finally, the ADCC activities (Fig. 2g) of each antibody were compared using Jurkat PD-1 cells. Commercial nivolumab showed significant ADCC activity, and WT IgG1 nivolumab showed the highest ADCC activity, as expected. Interestingly, the ADCC activities of both IgG1 and IgG4 nivolumab were significantly decreased by GD engineering and GD-IgG4 nivolumab showed the lowest ADCC among the antibodies. These data demonstrate that GD engineering decreased the effector function of the IgG4 backbone antibody such as nivolumab.

### Production of plant GD nivolumab in *N. benthamiana*

**Figure 3:**
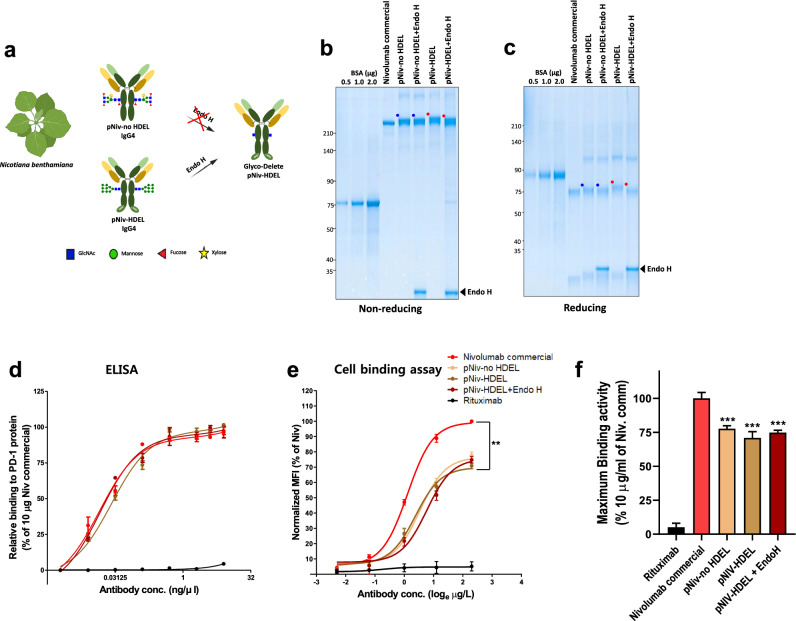
Production of plant GD nivolumab in *Nicotiana benthamiana*. (**a**) Schematic representation of plant nivolumab heavy chain and light chain produced in *N. benthamian*a leaves using the transient infiltration method. (**b**), (**c**) CHO-nivolumab, plant nivolumab no HDEL, plant-nivolumab-HDEL, and plant nivolumab with Endo H treatment were subjected to SDS-PAGE under non-reducing (**b**) and reducing (**c**) conditions. BSA (0.5 μg, 1.0 μg, and 2.0 μg) was used as the standard. (**d,e**) PD-1 binding affinity of antibodies (CHO-nivolumab, plant-nivolumab-HDEL, plant-nivolumab-HDEL with Endo H, and rituximab) were analyzed by two methods. (**d**) Binding to human PD-1 protein, as determined by ELISA. Two-fold dilutions of the antibodies were incubated on plates coated with the human PD-1 protein and detected with HRP-conjugated anti-human IgG antibody. (**e**) Cell surface PD-1 binding affinity was analyzed with PD-1–expressing CHO cells using FACS. Various concentrations (0.1 μg/mL, 0.3 μg/mL, 1 μg/mL, 3 μg/mL, and 10 μg/mL) of each antibody were used, with 2^nd^ anti-human IgG-FITC was used to detect each antibody. **(f)** The activity of each antibody at the maximum concentration (10 μg/ml) of each antibody was summarized. The mean fluorescence intensity (MFI) of FITC treated with nivolumab commercial (10 μg/mL) was normalized to 100%. The MFI of each antibody-treated cell was expressed as the relative %. ***p ≤ 0.001 compared to 10 μg/mL of nivolumab commercial. Rituximab was used as negative control in **(d)** and **(e,f)**.

Plant-nivolumab was produced in *N. benthamiana* via agrobacterium-mediated infiltration of the nivolumab light chain and heavy chain. HDEL, the ER retention tag was attached to the heavy chain of the IgG4 backbone, forming a high mannosidase glycan. High mannosidase glycan was cleaved by Endo H, a recombinant glycosidase yielding the GD form of the heavy chain glycan (Fig. 3a). No-HDEL tagged nivolumab was co-produced to prove the high mannose glycan selective cleavage of Endo H. First, their biochemical properties were confirmed using non-reducing and reducing SDS-PAGE (Fig. 3b,c), with commercial nivolumab used as a control. The reduction of molecular weight resulted by Endo H activity was only shown in plant nivolumab-HDEL, not in nivolumab-no-HDEL, confirmed that plant GD-nivolumab was successfully produced via in vitro enzyme reaction of Endo H from plant nivolumab HDEL. To analyze the binding affinity of plant GD-nivolumab, ELISA was performed using recombinant PD-1 protein (Fig. 3d). All forms of plant nivolumab including plant-GD nivolumab showed the same binding affinity to PD-1 protein as commercial nivolumab, as previously reported^29^. Their physiological binding was confirmed through the binding assay with PD-1 expressing CHO-K1 cells, using FACS (Fig. 3e). Interestingly, all plant nivolumab including plant GD nivolumab showed approximately 30% lower binding affinity compared to that of commercial nivolumab. Despite the reduced antigen binding capacity of the plant antibodies, this study demonstrated that GD antibodies can be produced in plants by an in vitro enzymatic reaction of HDEL-tagged heavy chains with Endo H.

### PD-1 blockade activity and ADCC efficacy of plant GD nivolumab

**Figure 4:**
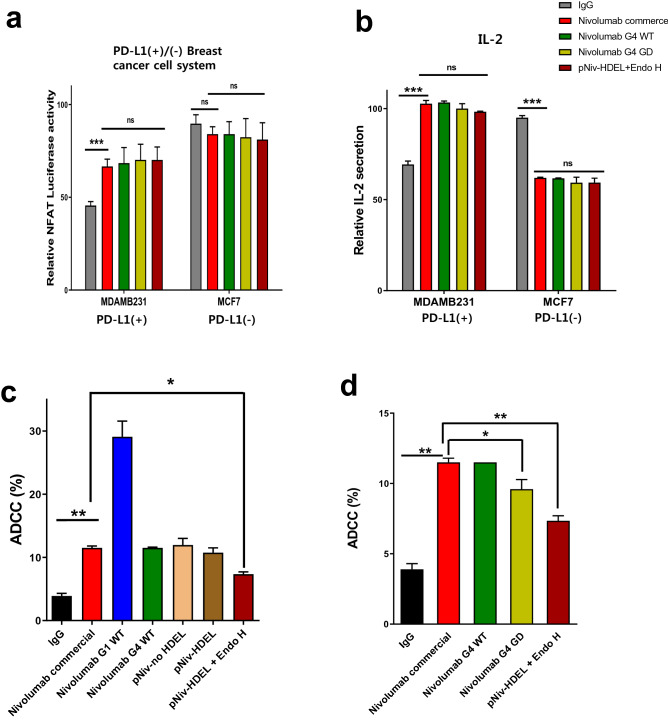
PD-1 blockade activity and ADCC efficacy of plant GD nivolumab. **(a**) PD-1 blockade activity of plant GD nivolumab was confirmed by the NFAT luciferase assay. Jurkat PD-1-NFAT cells were co-cultured with PD-L1 (+) or PD-L1 (−) cancer cells and stimulated with the anti-CD3/anti-CD28 activator. Each antibody was treated at a concentration of 30 μg/mL, and luciferase activity was measured 6 h after stimulation. **(b)** IL-2 secretion activity of T cells. Jurkat PD-1 cells were co-cultured with breast cancer cell lines and treated with the anti-CD3/anti-CD28 activator and 30 μg/mL of antibodies. After 24 h, the supernatants were collected, and the amount of secreted cytokine was determined by ELISA with IgG as a negative control. **(c)** The ADCC efficacy of plant GD nivolumab was analyzed using calcein-loaded Jurkat PD-1 cells and FACS. Nivolumab G1 WT and IgG was used as positive and negative control, respectively. Cell death rate was deducted from the percentage of live rate which was calculated by the numbers of calcein-positive cells 6 h after 10 μg/mL of each antibody treatment. The effector cell: target cell ratio was 3:1. **(d)** Comparison of ADCC between plant GD nivolumab and CHO-GD nivolumab. Statistical significances were indicated as *p ≤ 0.05, **p ≤ 0.01, and ***p ≤ 0.001, respectively. ns, not significant.

The PD-1 blockade function of plant GD-nivolumab and CHO GD-nivolumab were assessed through two different ways, Jurkat PD-1-NFAT luciferase reporter cell system and IL-2 cytokine release assay (Fig. 4a,b). Upon treatment with 30 μg/mL of each type of the nivolumab in PD-L1(+) cells, the luciferase activity of Jurkat PD-1 NFAT-luciferase stable cell and the IL-2 secretion of Jurkat PD-1 cells were increased. The increased magnitudes were similar for all forms of nivolumab including commercial nivolumab (Fig. 4a). In contrast, the function of PD-L1 (−) cells was not altered or decreased by any of the types of nivolumab, confirming that nivolumab specifically blocked the PD-1 and PD-L1 interaction (Fig. 4b). These data demonstrate that plant GD-nivolumab has the PD-1 blocking activity as based on the PD-1 and PD-L1 interaction. Finally, ADCC of plant GD-nivolumab was compared with that of CHO GD-nivolumab using Jurkat PD-1 cells (Fig. 4c). Plant nivolumab-HDEL with Endo H, which yielded plant GD nivolumab, exhibited reduced ADCC of plant nivolumab. Reduction in ADCC was also observed for CHO-GD nivolumab. These data indicated that plant GD engineering also reduced the ADCC of nivolumab without significantly altering the PD-1 blocking functions.

## Discussion

We constructed a GD CHO cell system and GD plant system by regulating the glycosylation process of CHO cells and plants to reduce ADCC of nivolumab. GD-nivolumab produced in both systems maintained its intrinsic anti-PD-1 function and significantly reduced T cell cytotoxicity. By retaining one sugar chain, GD provides structural stability and can produce a uniform antibody with the same sugar chains, maintaining the efficacy when used as a drug compared to when all sugar chains are removed.

Since blocking the interaction between PD-1 and PD-L1 is an important clinical strategy for the cancer treatment, various immune checkpoint inhibitors of different cells are currently being developed^1, 2^. Expression of PD-1 Is induced in different kind of cells such as T cells, NK cells, B lymphocytes, macrophages, DCs, and monocytes, thus, anti-PD-1 antibodies can be used as immune checkpoint modulators in these cells. As immune checkpoint modulators become more important, strategies for eliminating the ADCC of these antibodies are needed. In the case of anti-PD-1 antibody, FcγR binding affinity should be removed because it induces the death of the T cells and subsequently performance of the antibody is reduced due to the following reasons. First, IgG4 and IgG1 show similar levels of binding to FcγR1^9^. Although IgG4 has very low binding ability with FcγRIIIa expressed in natural killer cells, its binding ability with FcγRI expressed in macrophages, monocytes, DCs, and other cells is as high as that of IgG1. High affinity of IgG4 for these cells^30^ may induces cytotoxicity. Second, the drug efficacy may be reduced during the cancer treatment due to the blocking effect of IgG4^8^. If IgG4 first occupies the antibody receptor of immunogenic cells, the target antibody (usually IgG1) cannot bind to the immunogenic cells. In addition, considering the relationship between locally occurring antibodies and antibody receptors as well as the combination therapy of the target anticancer agent and immune checkpoint inhibitor^10^, a strategy for eliminating the ADCC of immune checkpoint inhibitors is mandatorily needed. GD can maximize the effect of combination therapy by removing the antibody receptor binding of the immune checkpoint inhibitory antibody. Recently, the “null effector” function of the Fc region has been developed for various applications. The L234F/L235E/P331S^31^ mutation prevents binding to FcγRs (including FcγRI, FcγRIIa, and FcγRIIIa) or C1q, or hybridization with IgG2/IgG1 as observed in BMS-986179^32^. Additional comparative studies are needed to determine the advantages and disadvantages of these technologies, including those of GD.

In glycan analysis, Endo T did not completely remove the high mannose residue in nivolumab (Supplementary Fig. S2, Supplementary Table S1). In general, the CHO system does not generate homogenous products when the glycan of antibodies is modified through glyco-engineering^33, 34^. Further processing of Endo H before the isolation process can be used to overcome this limitation. Transgenic plants are useful for GD antibody production because the final glycan structure of engineered transgenic plants is mostly homogeneous compared to that in the CHO system^35^. Although we failed to produce transgenic *Arabidopsis thaliana* because of the lethal effects of Endo T over-expression (data not shown), GD transgenic plant resistant to Endo T over-expression can likely generate uniform GD antibodies. In addition, the GD-nivolumab from CHO cells can be uniformly produced by performing Endo H treatment after purification.

As previously reported^29^, the binding affinity for PD-1 in ELISA system confirmed that there was no difference in binding affinity between CHO and plant antibodies (Fig. 3d). However, in cell expressed PD-1 binding assays, the binding affinity of plant derived nivolumab was significantly lower than that of the mammalian derived antibody (Fig. 3f). Ofatumumab^36^ and rituximab^37^, but not obinutuzumab^38^, showed the lower avidity than antibodies from CHO cells; in case of rituximab, the binding ability differed depending on the plant species that produced^37^. The cause of the different binding affinities in between the ELISA and cell binding assays remains unclear. Antibodies produced by applying the GD technique in the CHO system had no changes in the PD-1 binding (Fig. 1d,e). These results suggest that the function of the Fab of nivolumab is completely separated from the function of the Fc modified by GD. Therefore, GD technology can be applied to all types of IgG4 antibodies as long as the antibody does not contain a glycan in the Fab region. These results also show that the GD technology is a suitable platform for generating bio-betters as ADCC-null effector. In addition, the ADCC-reducing effect of GD was embodied in plant systems (Fig. 4c,d). These results s when an antibody such as an immunomodulatory checkpoint inhibitor is produced based on plant GD. In other words, the plant GD system not only removes plant-specific sugar chains, but also has the advantage of reducing the risk of target cell death, providing a good base technology for attempts to use plants as alternative production systems for biosimilar.

In conclusion, GD nivolumab was successfully produced in a CHO cell system and in *N. benthamiana* leaves. GD nivolumab produced in CHO cells and plants had low ADCC without any change in PD-1 inhibition functions. Therefore, the GD technology can be applied to various signal-regulating antibodies to improve their therapeutic effects.

## Methods

### Construction of GnTI knockout CHO cells and Endo T over-expression for GD CHO

The homology-independent targeted insertion (HITI CRISPR/Cas9 method^39^ was used to knockout GnTI in CHO cells (Korean Cell Line Bank). The sgRNA target region in exon 2 of the *MGAT1* (NCBI ENSG00000131446) gene (encoding the GnTI enzyme) was cleaved by transfected sgRNA and Cas9. The internal ribosomal entry site followed by the blasticidin S deaminase gene were inserted from a co-transfected donor plasmid at the cleavage site, causing knockout of the *MGAT1* gene and also marking the knockout cells (Supplementary Fig. S1a). The *MGAT1* knockout was confirmed via PCR and sequencing (Supplementary Fig. S1b). A lentivirus system was used to overexpress Endo T (NCBI LOC6044791)^40^ resulting in one GlcNAc in the N-glycan chain. Endo T overexpressed and GnTI knockout cells were selected by co-treatment with blasticidin S and hygromycin.

### Generation and purification of GD nivolumab with IgG1 and IgG4 backbone

Light and heavy chain sequences of nivolumab were obtained from GenBank (MC034325) and long chain cDNAs were synthesized by Bioneer. To produce IgG1 and IgG4 nivolumab, light and heavy chains of nivolumab with IgG1 and IgG4 backbone were transfected into HEK cells (ATCC, American Type Culture Collection) to generate lentiviruses. The HEK cell media containing lentiviruses were treated in CHO WT and GD CHO cells. Nivolumab-producing cells grown to 80% confluency were refreshed with EX-CELL® CD CHO Serum-Free medium (Sigma) containing 1 mM sodium butyrate. Conditioned media containing antibody was obtained by further incubation for 14 days at 30 °C in 5% CO_2_ incubator. The antibodies were collected and purified using a protein A column (Thermo Fisher Scientific). Buffer changes and sterilization were performed using an Amicon® Ultra-2 (UFC801024). The antibodies were analyzed using SDS-PAGE and coomassie blue staining, and their concentrations were quantified relative to the band intensities 0.5, 1.0, and 2.0 μg bovine serum albumin used as a standard.

### Production of plant GD nivolumab from *Nicotiana benthamiana*

Plant codon-optimized light and heavy chains were inserted into pCAMBIA 1300 binary vector (Supplementary Fig. S3a) with N-terminal BIP sequence (signal sequence for endoplasmic reticulum (ER) localization) and C-terminal with or without HDEL tag (ER retention signal sequence).

The constructs were transformed into *Agrobacterium tumefaciens* GV3101 competent cells using the freeze–thaw method. Transformed agrobacteria were incubated in YEB medium containing 50 mg/mL kanamycin and 50 mg/mL rifampicin at 28 °C for 2 days, then infiltrated into the abaxial side of leaves using a syringe. Four-week-old tobacco (*N. benthamiana L.*) plants, grown on the soil at 25 ± 0.5 °C under long-day conditions (16 h light and 8 h dark), were infiltrated and further incubated for 3–4 days under a 16 h light/8 h dark cycle at 25 ± 0.5 °C. The leaves harvested after 3–4 days after infiltration were ground under liquid nitrogen. Total soluble proteins were extracted with protein extraction buffer (50 mM Tris–HCl (pH 7.2), 150 mM NaCl, and protease inhibitor cocktail (Sigma-Aldrich)). The protein suspensions were centrifuged 3 times at 16,000×*g* for 30 min at 4 °C with Miracloth filters in between. The clarified extract was filtered through 0.22 μm pore filters and then loaded onto a protein A column (Thermo Fisher Scientific). The column was washed with extraction buffer, and antibodies were eluted using 100 mM glycine (pH 3.0), then immediately neutralized with 2.0 M Tris–HCl (pH 7.4). The antibody concentration was measured using a Human IgG ELISA Kit (E88-104, Bethyl Laboratories), and equal amounts of antibody used in each experiment were confirmed by the band intensity calculation after coomassie staining of antibodies in SDS-PAGE gel.

### Mass analysis for antibody

The molecular mass of the antibodies was determined via reversed-phase (RP) separation using Waters Acquity Iclass UPLC system (Milford). Separation was performed using a Thermo Fisher Scientific MabPac™ RP column (2.1 mm, 50 mm, 4 μm particle size) at a flow rate of 0.2 mL/min. The mobile phases were 0.1% formic acid in water (eluent A) and 0.1% formic acid in acetonitrile (eluent B) in gradient mode. The gradient applied over 0–2 min, fixed at 25% eluent B for 2–20 min, and increased linearly from 25 to 45% eluent B. The effluent was injected into an LTQ Orbitrap mass spectrometer (Thermo Fisher Scientific). The Fourier transform mass spectrometry resolution and mass range were 120,000 and 400–4000 m/z, respectively. The mass spectra were deconvoluted using Protein Deconvolution 2.0 in isotopically unresolved mode.

### Binding affinity test using FACS and PD-1 expressing cells

The binding ability of antibodies to recombinant PD-1 protein was evaluated using ELISA. Briefly, a MaxiSorp 96-well ELISA plate was coated with 10 ng/well (100 μL) of recombinant human PD-1 protein (#8986-PD-100, R&D Systems) at 4 °C overnight. A serial dilution of antibodies in PBS was incubated at 37 °C for 1 h, and then washed with PBS-T. Goat anti-human IgG-HRP was added and incubated at 37 °C for 1 h, then washed with PBS-T. The TMB substrate solution was added for coloring for 20 min and stop solution (2 M H_2_SO_4_) was added then the absorbance at 450 nm was determined using a Cytation™ reader (Bio-Tek). To compare the binding affinities of the antibodies to cell surface expressed PD-1, FACS was used. 1.5 × 10^5^ Jurkat (Korean Cell Line Bank) PD-1 cells were treated with antibodies (0.1, 0.3, 1, 3, and 10 µg/mL) for 30 min, followed by incubation with anti-human Ig Fc-specific FITC-conjugated secondary antibody for 30 min at 4 °C. Rituximab (Roche) was used as a negative control. Binding was measured as the geomean fluorescence intensity of each sample using FACS Verse (BD biosciences) and calculated using FlowJo software (TreeStar).

### Cytokine production test by ELISA

1 × 10^5^ cancer cells were seeded into a 96-well round-bottomed plate (Thermo Fisher Scientific), then 1 × 10^5^ Jurkat PD-1 cells were co-cultured. After 10 min, the cells were stimulated with 5 µL anti‑CD3/anti‑CD28 activator (25 IU, #10971, STEMCELL Technologies) and treated with each antibody (10 μg/mL) for 24 h at 37 °C CO_2_ incubator. The supernatant was collected by centrifuging the plate at 1500 rpm for 20 min. The levels of IL-2 and INF-γ were measure by ELIZA (#431081 and #430101, respectively, Biolegend). HEK 293 T cell (ATCC), breast cancer cell lines MCF7 (PD-L1 (−), Korean Cell Line bank), and MDAMB231 (PD-L1 (+), Korean Cell Line Bank) were used to observe anti-PD1 antibody activity dependent on PD-L1 expression.

### NFAT-luciferase reporter system for measuring PD1 inhibition

The Jurkat-PD-1 cell line was firstly developed by stable expression of human PD-1 by puromycin-resistant lentivirus system and high-level PD-1 expressed cells were sorted by FACS. Then Jurkat-PD-1-NFAT cell line was generated by stable co-expression of pGL3 luciferase vector under control of NFAT response elements from the IL-2 promoter (#17870, Addgene). Cancer cells (5 × 10^5^ cells) were seeded into a white 96-well plate and cultured at 37 °C and 5% CO_2_ for 12 h. After removing the medium, 1 × 10^6^ Jurkat-PD-1-NFAT luciferase cells in 50 μL medium was added. The cells were stimulated with 5 µL anti‑CD3/anti‑CD28 activator (25 IU, #10,971, STEMCELL Technologies) and treated with each antibody (30 μg/mL). The plate was incubated at 37 °C 5% CO_2_ incubator for 6 h and 100 μL luminescence substrate (Bio-Glo™ Luciferase Assay, Promega) was added, and relative luciferase units were scored using a SpectraMax®M5 luminometer (Molecular Devices).

### ADCC and CDC analysis

Peripheral blood mononuclear cells (PBMC) were purified from healthy donors who voluntarily participated in this study, for which informed consent was obtained to the study contents. All of these processes were conducted in accordance with the IRP procedure (#4-2016-0600) approved by the Yonsei University Institutional Review Committee. Briefly, 6 mL of blood and 6 mL PBS were loaded onto 6 mL Ficoll (Histopaque-1077, Sigma-Aldrich) and centrifuged at 400×*g* for 30 min at 20 °C to separate white blood cells. The white blood cell layer was collected and washed three times with RPMI-1640 medium to completely remove the platelets. To measure the survival rate of PD-1 expressing T cell in each cytotoxicity experiment, Jurkat-PD-1 cells were stained with 0.5 μM calcein-AM (C3100MP, Invitrogen) for 30 min at 37 °C to stain viable cells. 1 × 10^5^ cells were firstly treated 10 μg/mL of each antibody for 10 min at 37 °C CO_2_ incubator. After antibody treatment, PBMCs were added (PBMC: Jurkat PD-1 = 3:1) and incubated at 37 °C CO_2_ incubator for 4 h for ADCC measurement. In case of CDC measurement, 6 μL of rabbit complement MA (CL3221, Cedarlane) was added for 2 h at 37 °C CO_2_ incubator. The percentage of cell lysis (% of cells losing fluorescence among 1 × 10^4^ total cells counted) was calculated using FACS Verse and FlowJo software (v10.8.1).

### Statistical analysis

All statistical analyses were performed using GraphPad Prism software (version 5.0; GraphPad). To analyze the dose–response curves from the binding assay, the antibody concentrations were log-transformed, and binding affinity determined from the mean fluorescence intensities was normalized and analyzed through 4-parameter non-linear regression analysis (log (agonist) vs. normalized response—variable slope). ADCC data are presented as the means ± standard error of the mean. Statistical analysis was performed using Student’s *t*-tests, analysis of variance, followed by Tukey’s multiple comparison, or one-way analysis of variance. P values < 0.05 were considered statistically significant results.

### Regulatory and compliance

Experiments on plants in this work comply with the IUCN Policy Statement on Research Involving Species at Risk of Extinction and the Convention on the Trade in Endangered Species of Wild Fauna and Flora. The plants (Nicotiana benthamiana) and studies using these plants were approved by the Biosafety Committee of Yonsei University. Seeds and plants used in the experiments are not listed as threatened and were obtained from the publicly available seed company.

## Supplementary Information

Supplementary Figures.

Supplementary Table S1.

Supplementary Information.

## Data Availability

The datasets generated and/or analyzed during the current study are available in the Genebank. [nivolumab IgG1 heavy chain; OP142478, nivolumab IgG4 heavy chain; OP142479, nivolumab light chain; OP142477, nivolumab IgG4 heavy chain HDEL; OP142480, EndoT; OP142476].
