# Supplementary material for: Antibody-dependent cellular cytotoxicity-null effector developed using mammalian and plant GlycoDelete platform
Source: Sci Rep. 2022 Nov 8;12:19030. doi: 10.1038/s41598-022-23311-9 (PMC9643331; doi:10.1038/s41598-022-23311-9)
Supplement: Supplementary file 1 — Supplementary Figures. [file 41598_2022_23311_MOESM1_ESM.pdf]

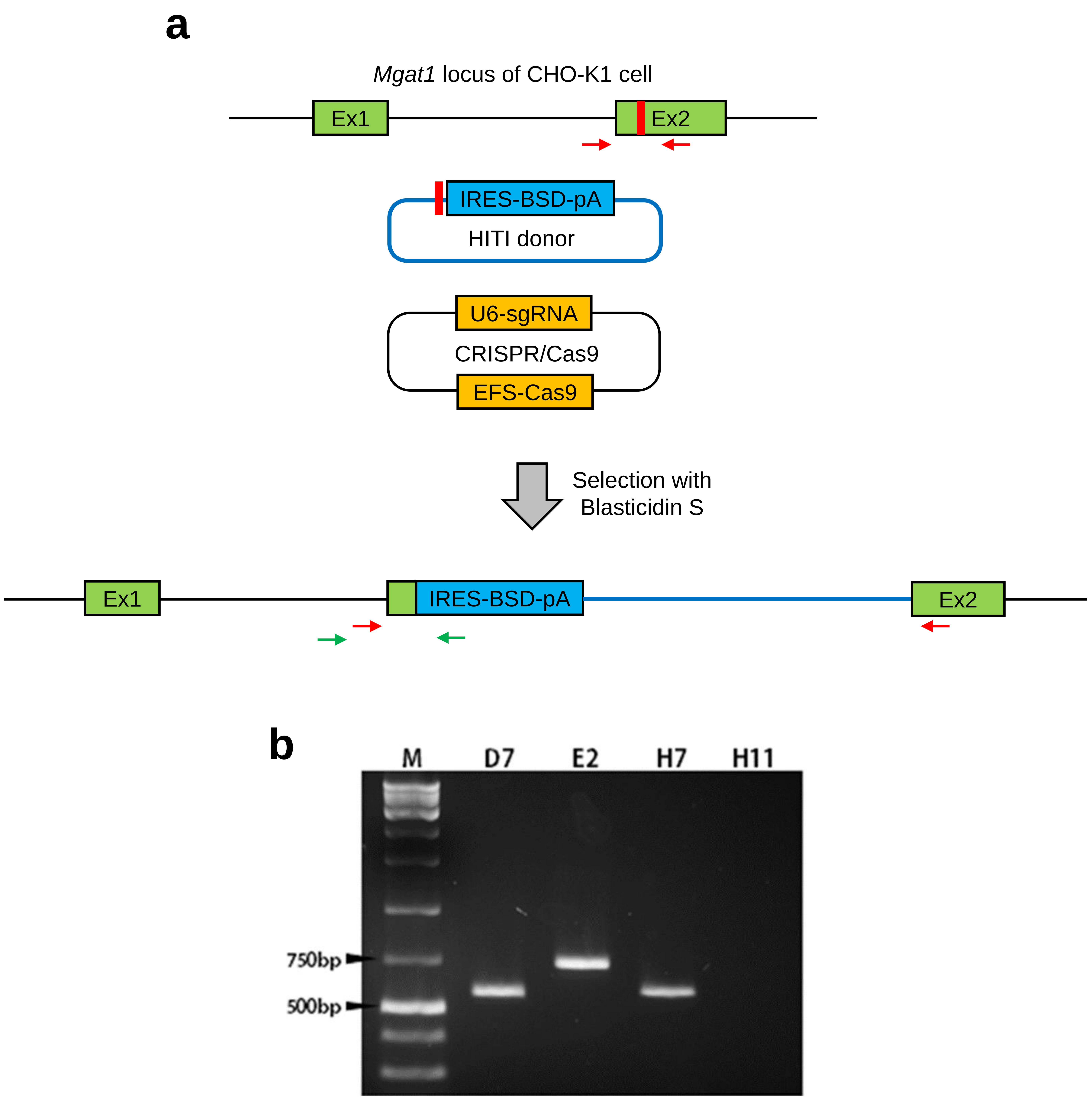

…ACGCTGAGGTGGAGTTGGAGCGGCAGCGGGGGCTGTTGCAGCA… : WT
…ACGCTGAGGTGGAGTTGGAGCGC---GGCGCTCCAACTCCACG… : H11

Supplementary Figure 1. Generation of CHO GnTI (-) cells. (**a**) Schematic presentation of CRISPR/Cas9-aided HITI. Donor and CRISPR/Cas9 plasmids were transfected. The donor plasmid was linearized by CRISPR/Cas9 in the cells and inserted into exon 2 of *MGAT1*. The red line is the target sequence of CRISPR/Cas9. (**b**) Validation of HITI. Genomic DNA was extracted from four cell clones following blasticidin S selection. The region including the CRISPR/Cas9 target was PCR-amplified using primer pairs (red arrows) followed by agarose gel electrophoresis. H11 clone did not amplify, suggesting successful HITI. The PCR product was then amplified with the green primer pair from H11 genomic DNA, sequenced, and aligned to the WT sequence. H11 clone showing indels (red) and inserted donor sequences (blue).

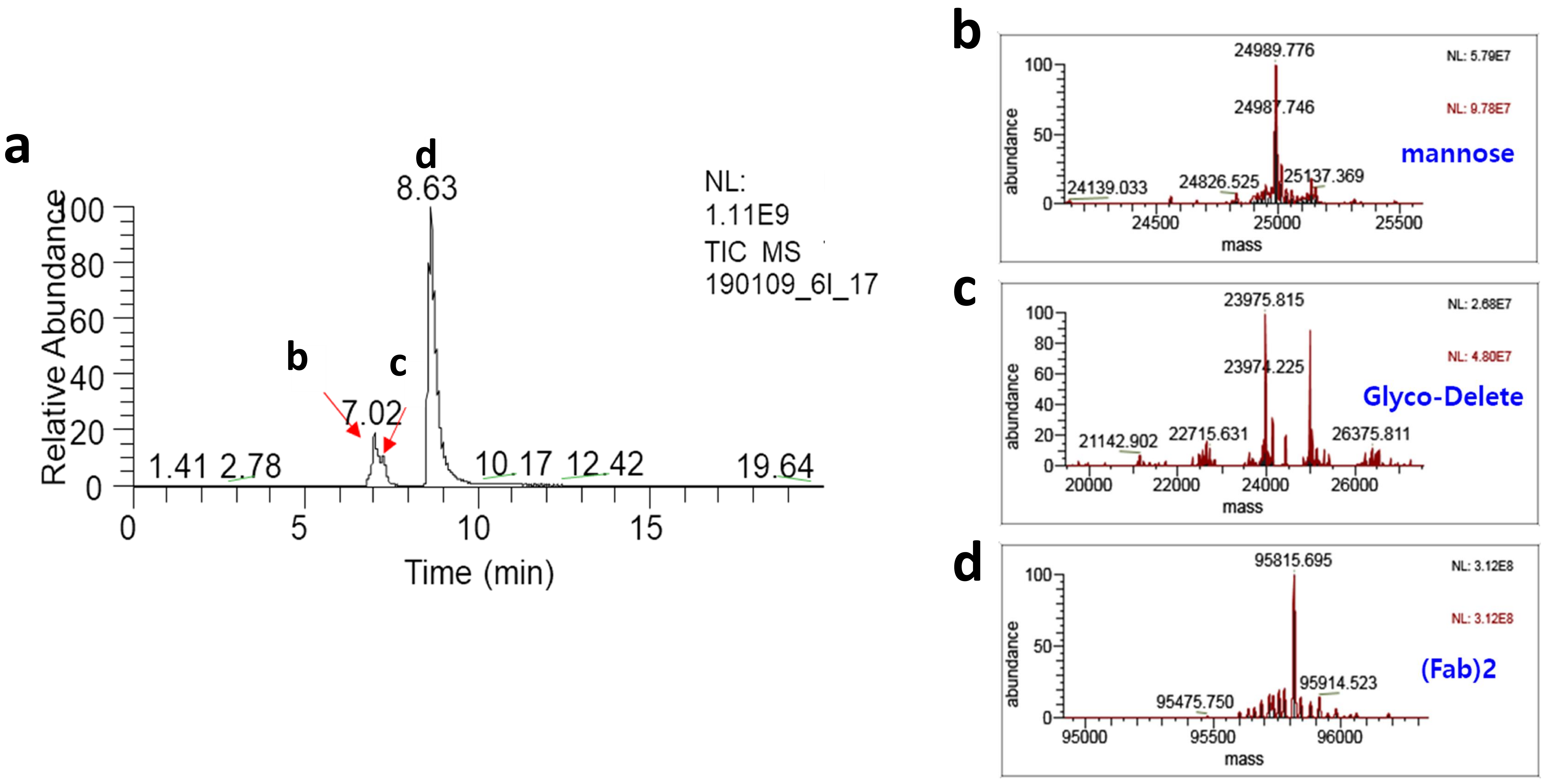

Supplementary Figure 2. Glycan analysis of IgG4 GD nivolumab from CHO cells. Total ion chromatogram (**a**) and deconvoluted spectrums (**b, c, d**) of the IgG4 CHO GD nivolumab after treatment with IdeS protease. Main peak indicating the calculated molecular mass of F(ab')$_2$(**d**) and Fc fragments. High-mannose form (**b**) and GD form (**c**) assembled together to form Fc fragments.

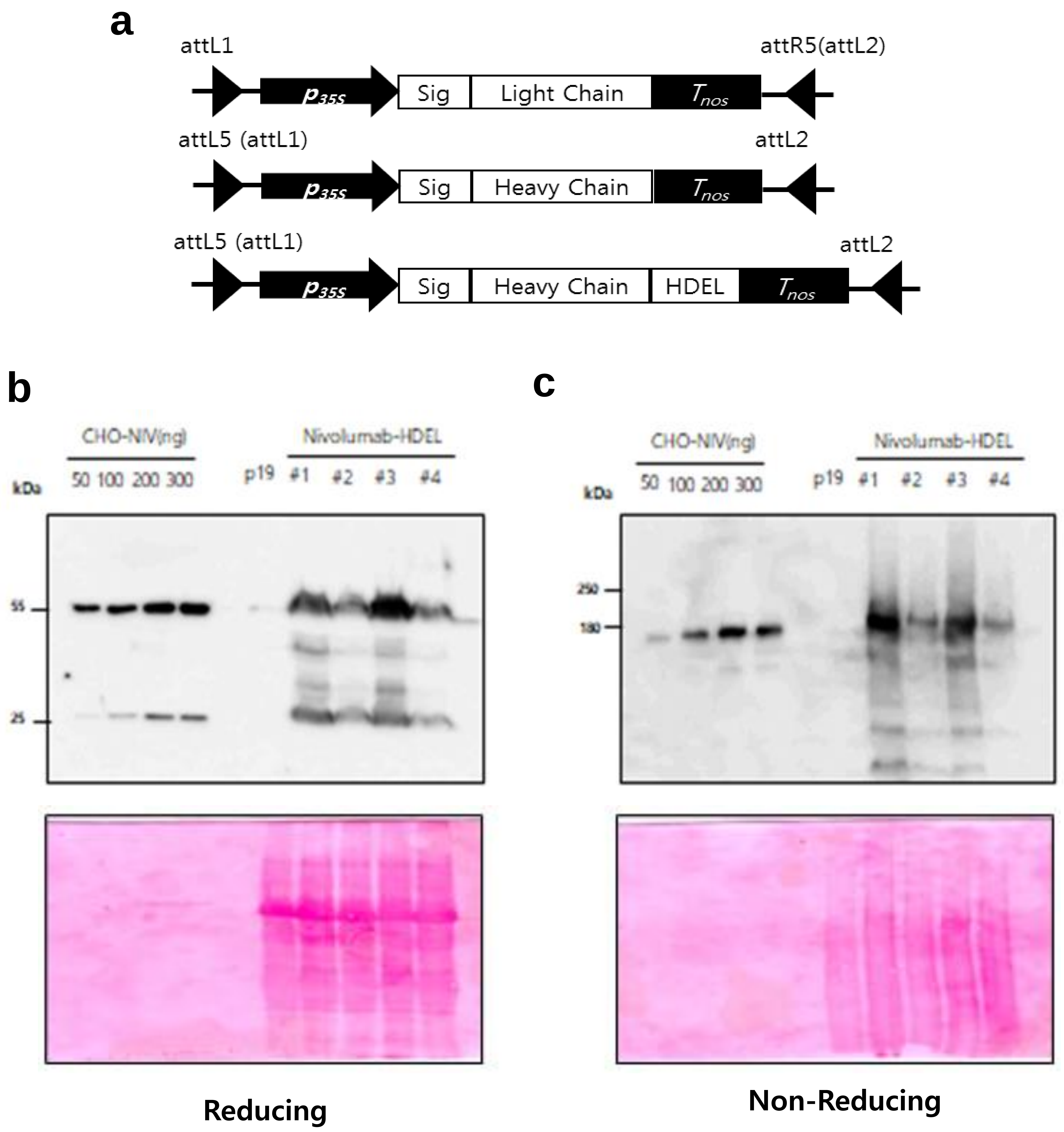

Supplementary Figure 3. Production of plant nivolumab in *Nicotiana benthamiana* (**a**) Construction of nivolumab light and heavy chains (no-HDEL tag and HDEL tag) for *N. benthamiana* genome incorporation (35S, cauliflower mosaic virus 35S promoter; Sig, BiP ER targeting signal sequence; HDEL, ER retention signal; NOS, nos terminator) (**b, c**) Western blot analysis for checking the expression of nivolumab-HDEL in *N. benthamiana*. (**b**) Reducing and (**c**) non-reducing conditions of SDS-PAGE were used to verify the integrity of the heavy and light chain proteins and heterodimer complexes of nivolumab, respectively. The expression levels of plant nivolumab were estimated using a concentration gradient of commercial nivolumab. Ponceau S–stained pink blots show the similar protein loading amounts of plant lysates.
