# Supplementary material for: Antibody-dependent cellular cytotoxicity-null effector developed using mammalian and plant GlycoDelete platform
Source: Sci Rep. 2022 Nov 8;12:19030. doi: 10.1038/s41598-022-23311-9 (PMC9643331; doi:10.1038/s41598-022-23311-9)
Supplement: Supplementary file 2 — Supplementary Table S1. [file 41598_2022_23311_MOESM2_ESM.pdf]

Supplementary Table S1. Intact mass analysis for glycan structure of CHO glyco-delete IgG4 nivolumab

CHOK1-Glycodeleted-H11-Nivol IgG4							
		Theoretical mass G0F-2Lys	Theoretical mass drug bank G0F-2Lys	Observed mass	△ mass	△ mass drug bank
1.Native		146235.13	146255.21	145752.20	**482.93**	**503.01**
2.Ides treate	Fc/2	25216.10	25216.10	24989.80	**226.30**	**226.30**
		25216.10	25216.10	23975.80	**1240.30**	**1240.30**
	F(ab')2	95838.97	95859.05	95815.70	23.27	43.35
