# Supplementary material for: Antibody-dependent cellular cytotoxicity-null effector developed using mammalian and plant GlycoDelete platform
Source: Sci Rep. 2022 Nov 8;12:19030. doi: 10.1038/s41598-022-23311-9 (PMC9643331; doi:10.1038/s41598-022-23311-9)

< Figure 1b Original gel image >

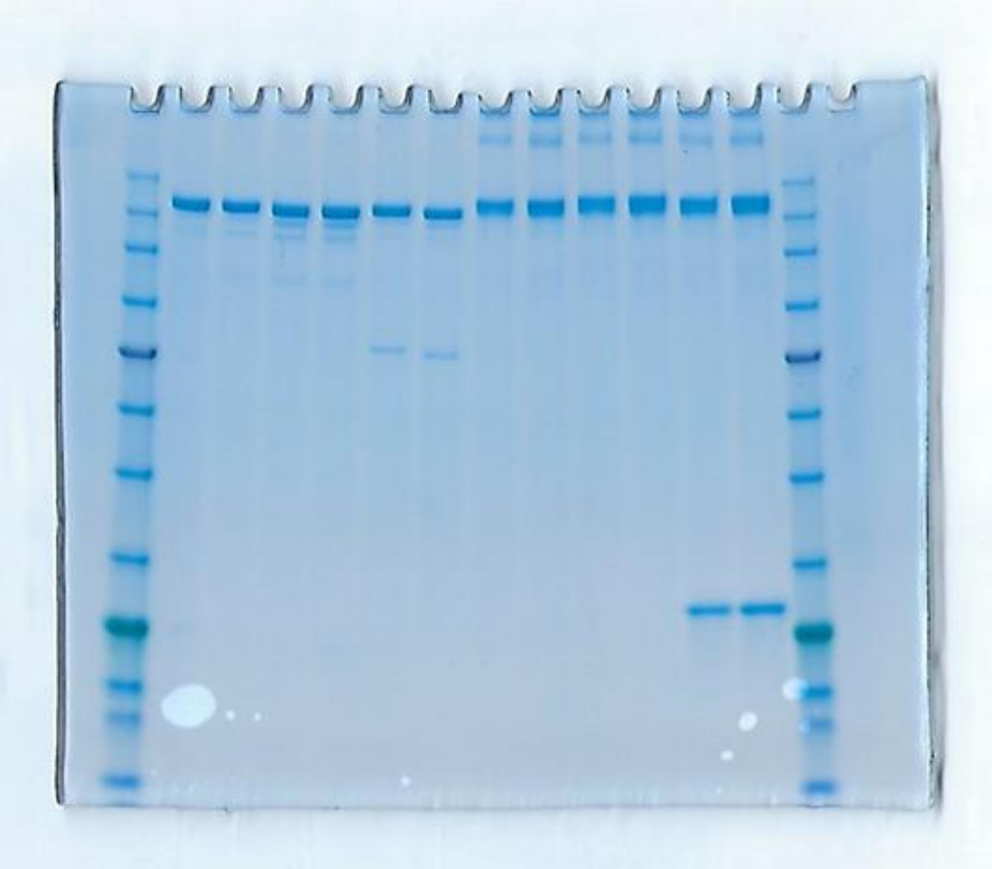

1 : Size marker
2 : Niv.-commercial
3 : Nivol G1 WT
4 : Nivol G1 GD1
5 : Nivol G1 GD2
6 : Niv G4 WT
7 : pNiv-noHDEL
8 : pNiv-noHDEL
9 : pNiv-HDEL
10 :pNiv-HDEL
11 : pNiv-HDEL+EndoH
12 : pNiv-HDEL+EndoH

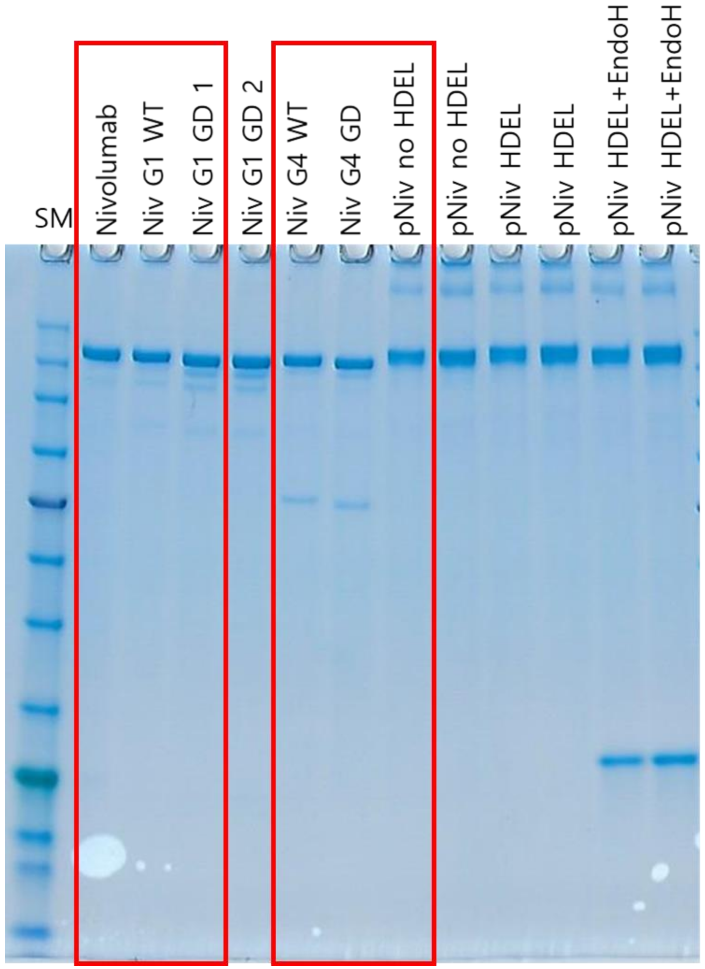

* Lane 2, 3, 4 and 6, 7, 8 were shown in Figure 1b

< Figure 1c Original gel image>

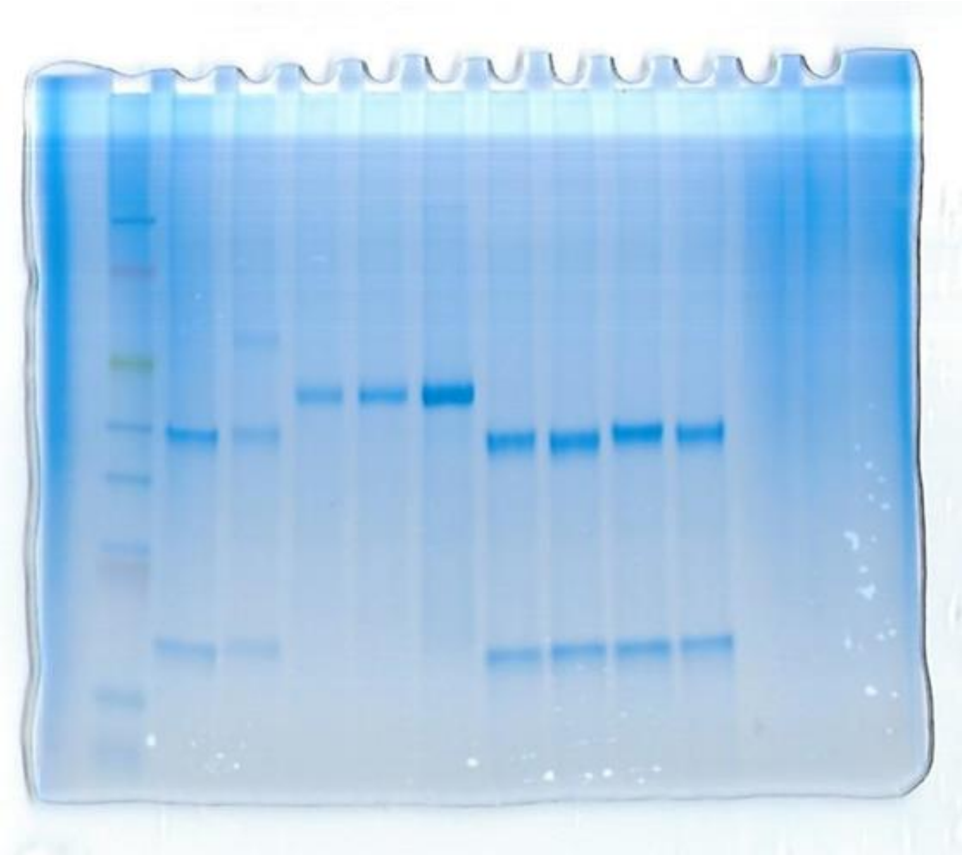

1 : Size marker
2 : Niv.-commercial
3 : pNiv-noHDEL
4 : BSA 0.5 ug
5 : BSA 1.0 ug
6 : BSA 2.0 ug
7 : Nivol G1 WT
8 : Nivol G1 GD
9 : Niv G4 WT
10 :Niv G4 GD
11: None
12: None

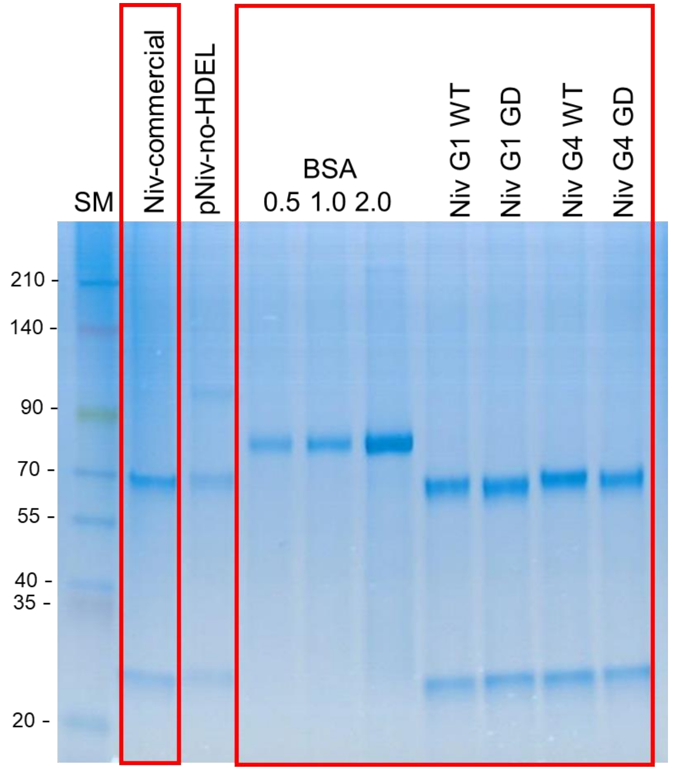

< Reducing PAGE condition>

* Lane 2, and 4~10 were shown in Figure 1C

< Figure 3b Original gel image >

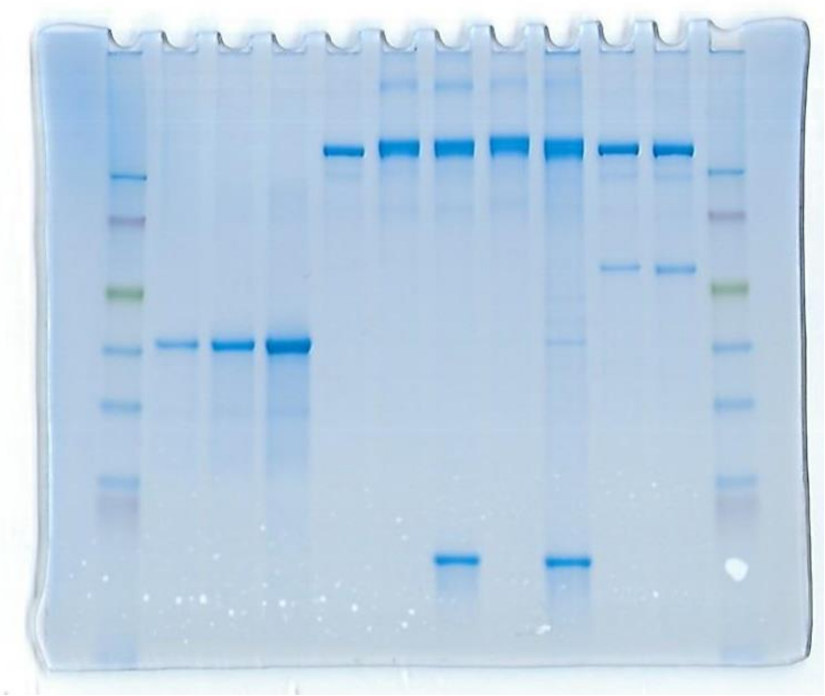

1 : Size marker
2 : BSA 0.5 ug
3 : BSA 1.0 ug
4 : BSA 2.0 ug
5 : CHO-Nivolumab
6 : plant-Nivol no HDEL
7 : plant-Nivol no HDEL + Endo H
8 : plant-Nivol HDEL
9 : plant-Nivol HDEL + Endo H
10 : Niv G4 WT
11 : Niv G4 GD
12 : Size marker

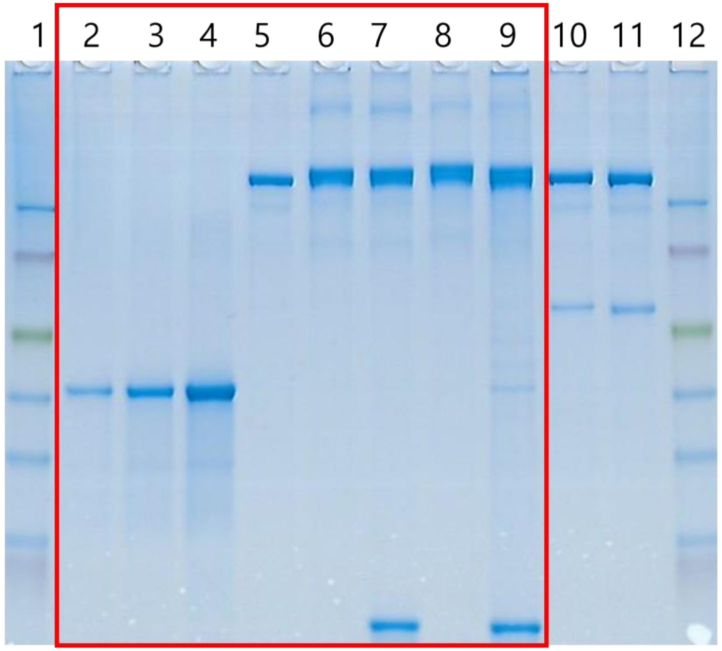

< non-reducing PAGE condition>

* Lane 2~9 were shown in Figure 3b

< Figure 3c Original gel image >

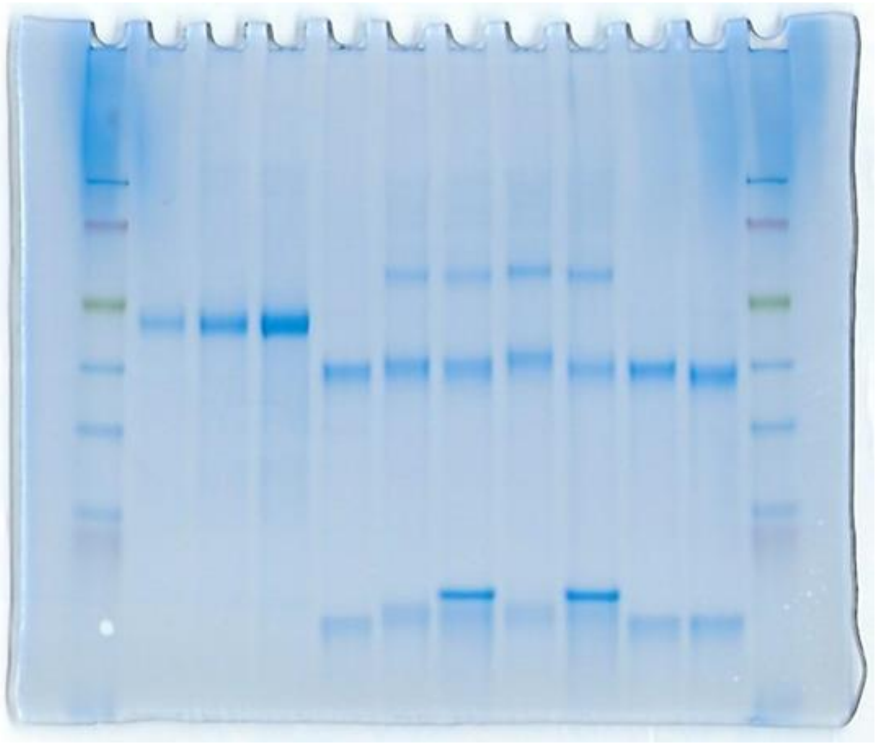

1 : BSA 0.5 ug
2 : BSA 1.0 ug
3 : BSA 2.0 ug
4 : CHO-Nivolumab
5 : plant-Nivol no HDEL
6 : plant-Nivol no HDEL + Endo H
7 : plant-Nivol HDEL
8 : plant-Nivol HDEL + Endo H
9 : Niv G4 WT
10 : Niv G4 GD

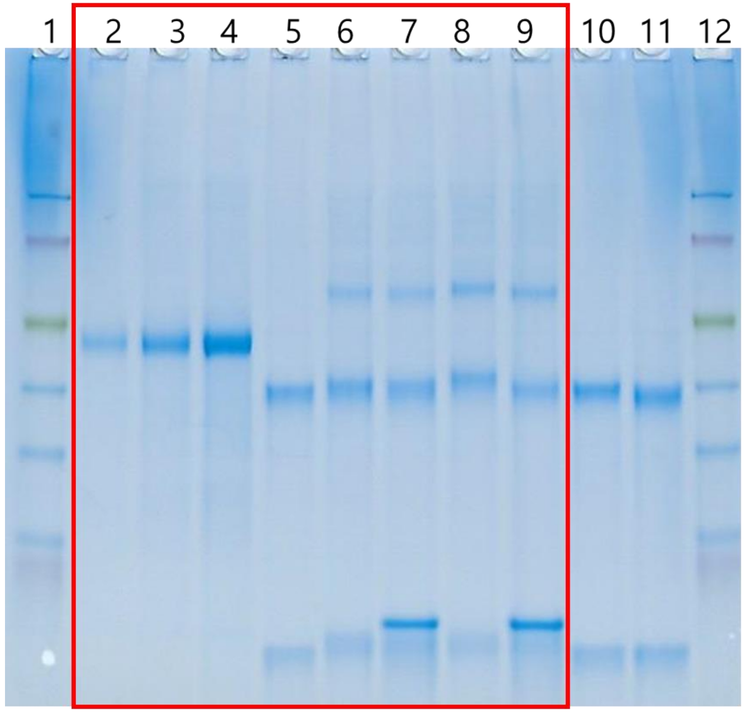

< reducing PAGE condition >

* Lane 2~9 were shown in Figure 3c

< Supplementary Figure 1 Original gel image >

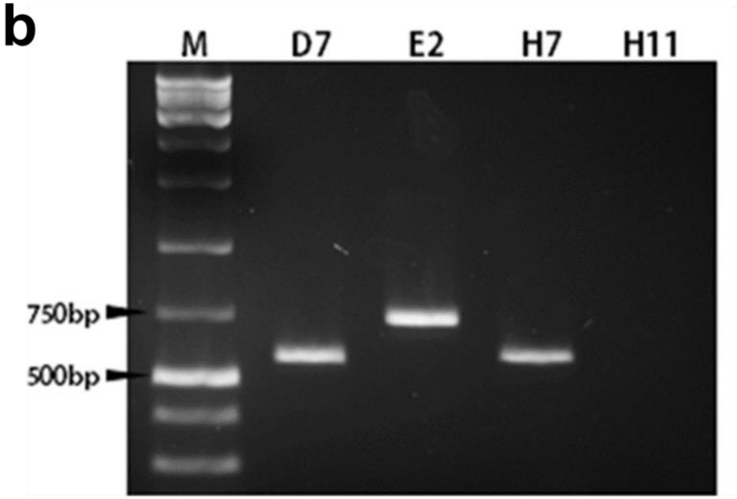

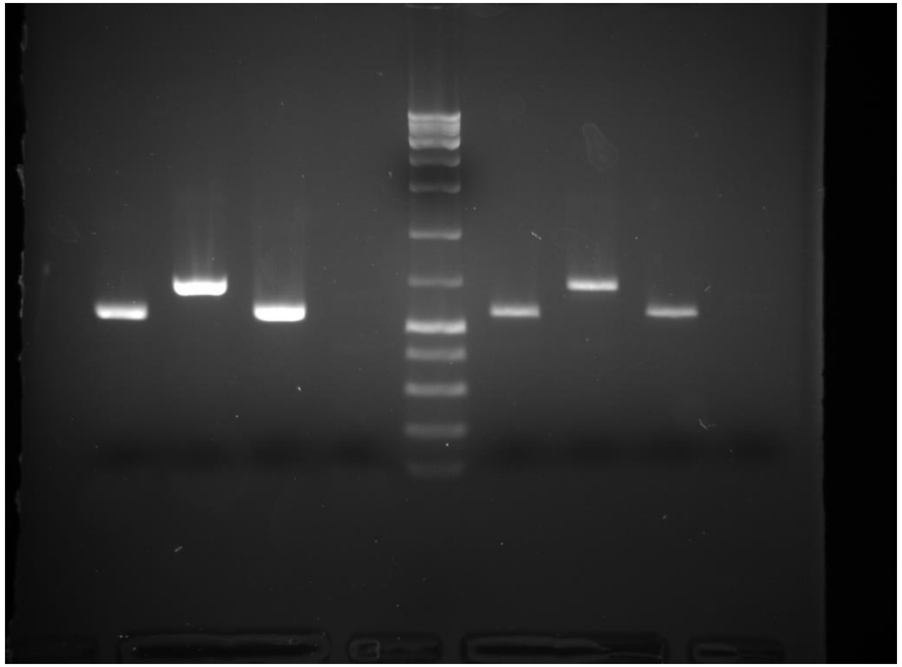

...ACGCTGAGGTGGAGTTGGAGCGGCAGCGGGGGCTGTTGCAGCA... : WT
...ACGCTGAGGTGGAGTTGGAGCGC---GGCGCTCCAACTCCACG... : H11

< Supplementary Figure 3b, c Original gel image >

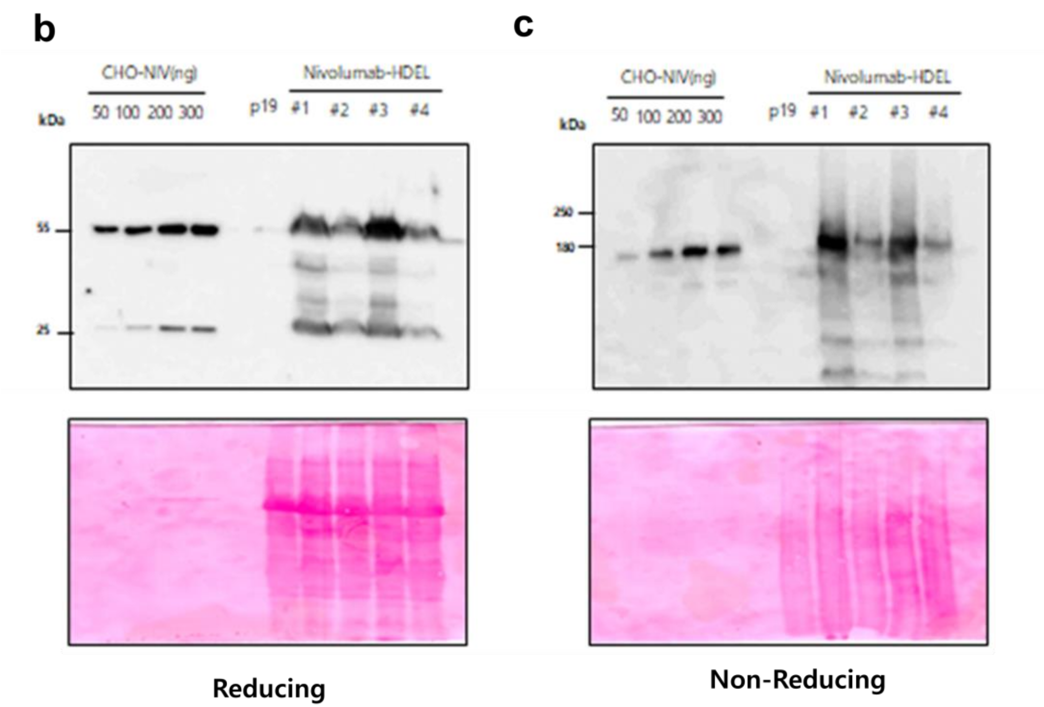

b

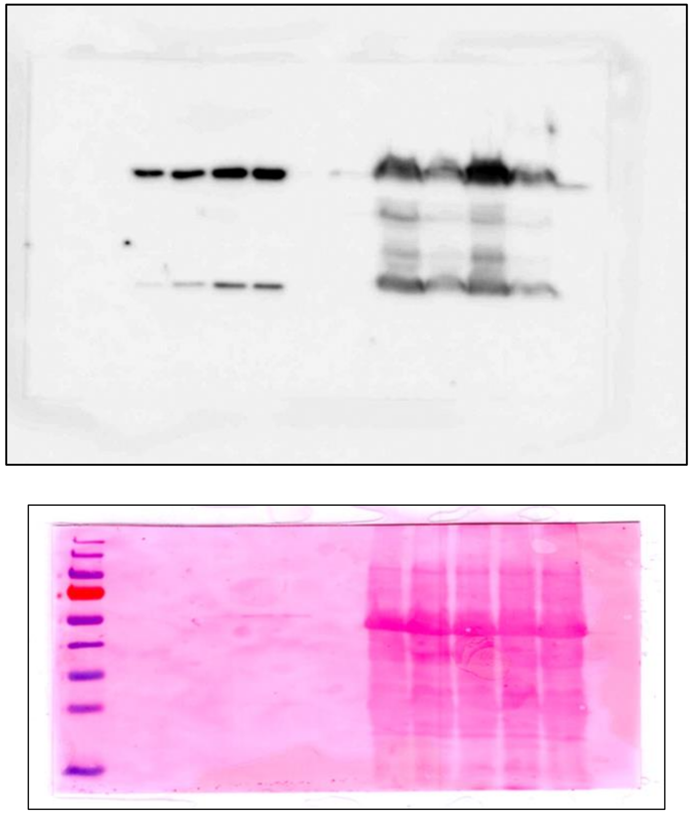

c

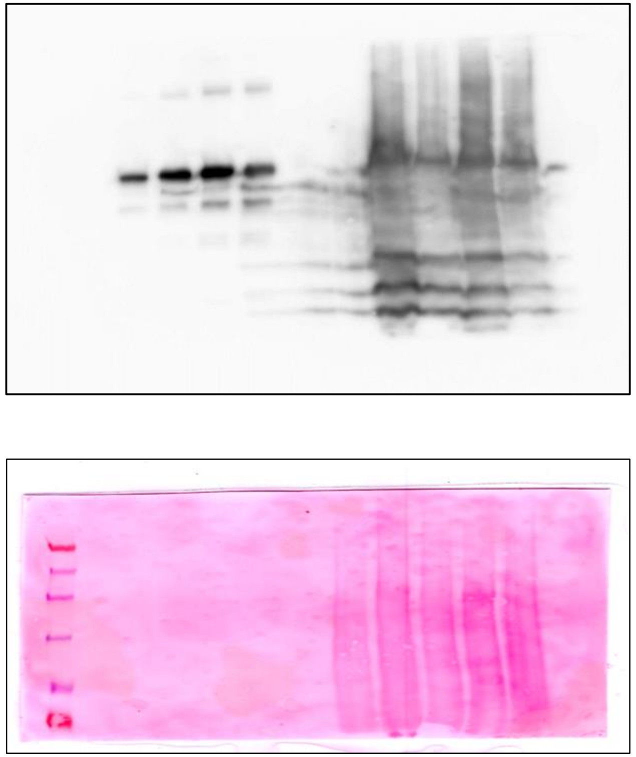

Supplement: Supplementary file 3 — Supplementary Information. [file 41598_2022_23311_MOESM3_ESM.pdf]
